# Supplementary material for: A fault-intrusion-tolerant system and deadline-aware algorithm for scheduling scientific workflow in the cloud
Source: PeerJ Comput Sci. 2021 Nov 2;7:e747. doi: 10.7717/peerj-cs.747 (PMC8576554; doi:10.7717/peerj-cs.747)
Supplement: Supplemental Information 6 [file peerj-cs-07-747-s006.zip › Myproject1.1/docs/org/cloudbus/cloudsim/network/datacenter/Switch.html]

Switch


JavaScript is disabled on your browser.


- Overview
- Package
- Class
- Use
- Tree
- Deprecated
- Index
- Help

- Prev Class
- Next Class

- Frames
- No Frames

- All Classes

- Summary:
- Nested |
- Field |
- Constr |
- Method

- Detail:
- Field |
- Constr |
- Method


org.cloudbus.cloudsim.network.datacenter

## Class Switch

- java.lang.Object
- - org.cloudbus.cloudsim.core.SimEntity
  - - org.cloudbus.cloudsim.network.datacenter.Switch

- All Implemented Interfaces:
  :   java.lang.Cloneable

  Direct Known Subclasses:
  :   AggregateSwitch, EdgeSwitch, RootSwitch

  ---

    

  ```
  public class Switch
  extends SimEntity
  ```

- - ### Field Summary

    Fields

    | Modifier and Type | Field and Description |
    | --- | --- |
    | `java.util.List<Vm>` | `BagofTaskVm` |
    | `int` | `datacenterid` |
    | `NetworkDatacenter` | `dc` |
    | `double` | `downlinkbandwidth` |
    | `java.util.List<Switch>` | `downlinkswitches` |
    | `java.util.Map<java.lang.Integer,java.util.List<NetworkPacket>>` | `downlinkswitchpktlist` |
    | `java.util.SortedMap<java.lang.Double,java.util.List<NetworkHost>>` | `fintimelistHost` |
    | `java.util.SortedMap<java.lang.Double,java.util.List<NetworkVm>>` | `fintimelistVM` |
    | `java.util.Map<java.lang.Integer,NetworkHost>` | `hostlist` |
    | `int` | `id` |
    | `double` | `latency` |
    | `int` | `level` |
    | `double` | `numport` |
    | `java.util.Map<java.lang.Integer,java.util.List<NetworkPacket>>` | `packetTohost` |
    | `java.util.ArrayList<NetworkPacket>` | `pktlist` |
    | `double` | `switching_delay` |
    | `double` | `uplinkbandwidth` |
    | `java.util.List<Switch>` | `uplinkswitches` |
    | `java.util.Map<java.lang.Integer,java.util.List<NetworkPacket>>` | `uplinkswitchpktlist` |
    | `java.util.Map<java.lang.Integer,NetworkVm>` | `Vmlist` |

    - ### Fields inherited from class org.cloudbus.cloudsim.core.SimEntity

      `FINISHED, HOLDING, RUNNABLE, WAITING`
  - ### Constructor Summary

    Constructors

    Constructor and Description

    `Switch(java.lang.String name,
    int level,
    NetworkDatacenter dc)`
  - ### Method Summary

    Methods

    | Modifier and Type | Method and Description |
    | --- | --- |
    | `protected java.util.List<NetworkHost>` | `getfreehostlist(int numhost)` |
    | `protected java.util.List<NetworkVm>` | `getfreeVmlist(int numVMReq)` |
    | `protected NetworkHost` | `getHostwithVM(int vmid)` |
    | `void` | `processEvent(SimEvent ev)` This method is invoked by the `Simulation` class whenever there is an event in the deferred queue, which needs to be processed by the entity. |
    | `protected void` | `processhostpacket(SimEvent ev)` |
    | `protected void` | `processpacket_down(SimEvent ev)` |
    | `protected void` | `processpacket_up(SimEvent ev)` |
    | `protected void` | `processpacket(SimEvent ev)` |
    | `protected void` | `processpacketforward(SimEvent ev)` |
    | `void` | `shutdownEntity()` This method is invoked by the `Simulation` before the simulation finishes. |
    | `void` | `startEntity()` This method is invoked by the `Simulation` class when the simulation is started. |

    - ### Methods inherited from class org.cloudbus.cloudsim.core.SimEntity

      `cancelEvent, clone, getEventBuffer, getId, getName, getNextEvent, getNextEvent, getState, numEventsWaiting, numEventsWaiting, pause, run, schedule, schedule, schedule, schedule, scheduleFirst, scheduleFirst, scheduleFirst, scheduleFirst, scheduleFirstNow, scheduleFirstNow, scheduleFirstNow, scheduleFirstNow, scheduleNow, scheduleNow, scheduleNow, scheduleNow, selectEvent, send, send, send, send, sendNow, sendNow, sendNow, sendNow, setEventBuffer, setId, setState, waitForEvent`
    - ### Methods inherited from class java.lang.Object

      `equals, finalize, getClass, hashCode, notify, notifyAll, toString, wait, wait, wait`

- - ### Field Detail


    - #### id

      ```
      public int id
      ```


    - #### level

      ```
      public int level
      ```


    - #### datacenterid

      ```
      public int datacenterid
      ```


    - #### uplinkswitchpktlist

      ```
      public java.util.Map<java.lang.Integer,java.util.List<NetworkPacket>> uplinkswitchpktlist
      ```


    - #### downlinkswitchpktlist

      ```
      public java.util.Map<java.lang.Integer,java.util.List<NetworkPacket>> downlinkswitchpktlist
      ```


    - #### hostlist

      ```
      public java.util.Map<java.lang.Integer,NetworkHost> hostlist
      ```


    - #### uplinkswitches

      ```
      public java.util.List<Switch> uplinkswitches
      ```


    - #### downlinkswitches

      ```
      public java.util.List<Switch> downlinkswitches
      ```


    - #### packetTohost

      ```
      public java.util.Map<java.lang.Integer,java.util.List<NetworkPacket>> packetTohost
      ```


    - #### uplinkbandwidth

      ```
      public double uplinkbandwidth
      ```


    - #### downlinkbandwidth

      ```
      public double downlinkbandwidth
      ```


    - #### latency

      ```
      public double latency
      ```


    - #### numport

      ```
      public double numport
      ```


    - #### dc

      ```
      public NetworkDatacenter dc
      ```


    - #### fintimelistHost

      ```
      public java.util.SortedMap<java.lang.Double,java.util.List<NetworkHost>> fintimelistHost
      ```


    - #### fintimelistVM

      ```
      public java.util.SortedMap<java.lang.Double,java.util.List<NetworkVm>> fintimelistVM
      ```


    - #### pktlist

      ```
      public java.util.ArrayList<NetworkPacket> pktlist
      ```


    - #### BagofTaskVm

      ```
      public java.util.List<Vm> BagofTaskVm
      ```


    - #### switching\_delay

      ```
      public double switching_delay
      ```


    - #### Vmlist

      ```
      public java.util.Map<java.lang.Integer,NetworkVm> Vmlist
      ```
  - ### Constructor Detail


    - #### Switch

      ```
      public Switch(java.lang.String name,
            int level,
            NetworkDatacenter dc)
      ```
  - ### Method Detail


    - #### startEntity

      ```
      public void startEntity()
      ```

      **Description copied from class: `SimEntity`**

      This method is invoked by the `Simulation` class when the simulation is started. This
      method should be responsible for starting the entity up.

      **Specified by:**
      :   `startEntity` in class `SimEntity`


    - #### processEvent

      ```
      public void processEvent(SimEvent ev)
      ```

      **Description copied from class: `SimEntity`**

      This method is invoked by the `Simulation` class whenever there is an event in the
      deferred queue, which needs to be processed by the entity.

      **Specified by:**
      :   `processEvent` in class `SimEntity`

      Parameters:
      :   `ev` - the event to be processed by the entity


    - #### processhostpacket

      ```
      protected void processhostpacket(SimEvent ev)
      ```


    - #### processpacket\_down

      ```
      protected void processpacket_down(SimEvent ev)
      ```


    - #### processpacket\_up

      ```
      protected void processpacket_up(SimEvent ev)
      ```


    - #### processpacket

      ```
      protected void processpacket(SimEvent ev)
      ```


    - #### processpacketforward

      ```
      protected void processpacketforward(SimEvent ev)
      ```


    - #### getHostwithVM

      ```
      protected NetworkHost getHostwithVM(int vmid)
      ```


    - #### getfreeVmlist

      ```
      protected java.util.List<NetworkVm> getfreeVmlist(int numVMReq)
      ```


    - #### getfreehostlist

      ```
      protected java.util.List<NetworkHost> getfreehostlist(int numhost)
      ```


    - #### shutdownEntity

      ```
      public void shutdownEntity()
      ```

      **Description copied from class: `SimEntity`**

      This method is invoked by the `Simulation` before the simulation finishes. If you want
      to save data in log files this is the method in which the corresponding code would be placed.

      **Specified by:**
      :   `shutdownEntity` in class `SimEntity`


- Overview
- Package
- Class
- Use
- Tree
- Deprecated
- Index
- Help

- Prev Class
- Next Class

- Frames
- No Frames

- All Classes

- Summary:
- Nested |
- Field |
- Constr |
- Method

- Detail:
- Field |
- Constr |
- Method
